# Supplementary material for: The causal relationship between the human gut microbiota and pyogenic arthritis: a Mendelian randomization study
Source: Front Cell Infect Microbiol. 2024 Nov 26;14:1452480. doi: 10.3389/fcimb.2024.1452480 (PMC11629706; doi:10.3389/fcimb.2024.1452480)
Supplement: Supplementary file 1 [file DataSheet1.zip › mendelian test/ebi-a-GCST90027833.csv_leave_one_out.pdf]

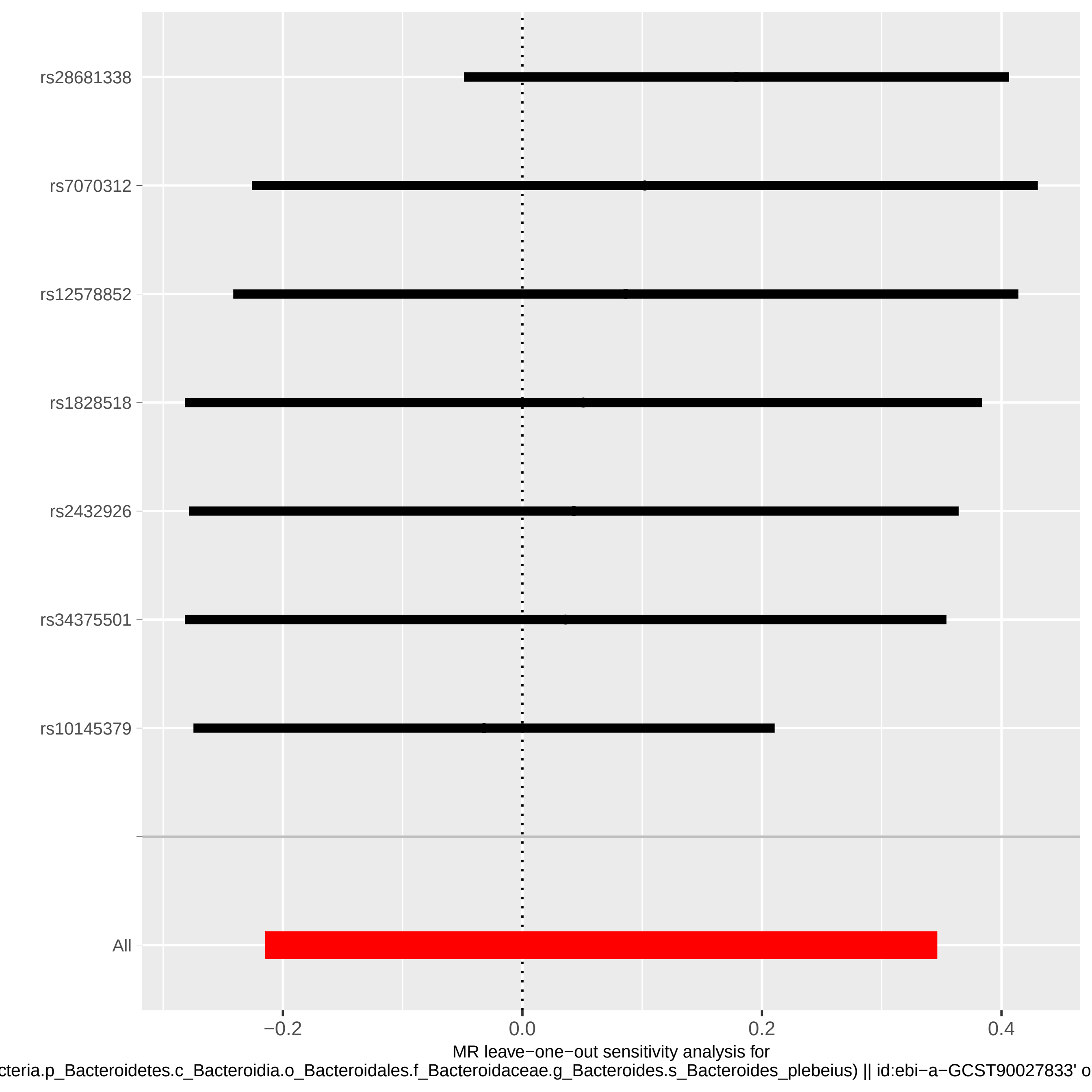
rs28681338
rs7070312
rs12578852
rs1828518
rs2432926
rs34375501
rs10145379
All
−0.2
0.0
0.2
0.4
MR leave−one−out sensitivity analysis for
cteria.p_Bacteroidetes.c_Bacteroidia.o_Bacteroidales.f_Bacteroidaceae.g_Bacteroides.s_Bacteroides_plebeius) || id:ebi−a−GCST90027833' o
